# Autonomic Effects of Controlled Fine Particulate Exposure in Young Healthy Adults: Effect Modification by Ozone

**DOI:** 10.1289/ehp.0900541

**Published:** 2009-04-24

**Authors:** Asghar A. Fakhri, Ljubomir M. Ilic, Gregory A. Wellenius, Bruce Urch, Frances Silverman, Diane R. Gold, Murray A. Mittleman

**Affiliations:** 1 Cardiovascular Epidemiology Research Unit, Department of Medicine, Beth Israel Deaconess Medical Center, Harvard Medical School, Boston, Massachusetts, USA; 2 Department of Medicine, Mount Auburn Hospital, Cambridge, Massachusetts, USA; 3 Gage Occupational and Environmental Health Unit, St. Michael’s Hospital, Toronto, Ontario, Canada; 4 Institute of Medical Science and; 5 Dalla Lana School of Public Health and Department of Medicine, University of Toronto, Toronto, Ontario, Canada; 6 Channing Laboratory, Department of Medicine, Brigham and Women’s Hospital, Harvard Medical School, Boston, Massachusetts, USA; 7 Department of Epidemiology, Harvard School of Public Health, Boston, Massachusetts, USA

**Keywords:** asthma, blood pressure, heart rate variability, ozone, particulate matter

## Abstract

**Background:**

Human controlled-exposure studies have assessed the impact of ambient fine particulate matter on cardiac autonomic function measured by heart rate variability (HRV), but whether these effects are modified by concomitant ozone exposure remains unknown.

**Objective:**

In this study we assessed the impact of O_3_ and particulate matter exposure on HRV in humans.

**Methods:**

In a crossover design, 50 subjects (19–48 years of age) were randomized to 2-hr controlled exposures to filtered air (FA), concentrated ambient particles (CAPs), O_3_, or combined CAPs and ozone (CAPs + O_3_). The primary end point was change in HRV between the start and end of exposure. Secondary analyses included blood pressure (BP) responses, and effect modification by asthmatic status.

**Results:**

Achieved mean CAPs and O_3_ exposure concentrations were 121.6 ± 48.0 μg/m^3^ and 113.9 ± 6.6 ppb, respectively. In a categorical analysis, exposure had no consistent effect on HRV indices. However, the dose–response relationship between CAPs mass concentration and HRV indices seemed to vary depending on the presence of O_3_. This heterogeneity was statistically significant for the low-frequency component of HRV (*p* = 0.02) and approached significance for the high-frequency component and time-domain measures of HRV. Exposure to CAPs + O_3_ increased diastolic BP by 2.0 mmHg (SE, 1.2; *p* = 0.02). No other statistically significant changes in BP were observed. Asthmatic status did not modify these effects.

**Conclusion:**

The potentiation by O_3_ of CAPs effects on diastolic BP and possibly HRV is of small magnitude in young adults. Further studies are needed to assess potential effects in more vulnerable populations.

Ambient air pollution has gained recognition as an important source of cardiovascular morbidity and mortality (Brook et al. 2004; Dockery et al. 1993; Pope et al. 1995). In addition to long-term mortality, daily fluctuations in fine particles [particulate matter with median aerodynamic diameter < 2.5 μm (PM_2.5_)] and ozone have been linked to short-term cardiovascular and pulmonary mortality (Bell et al. 2004; Brook et al. 2004; Dominici et al. 2006). Studies in subjects with implanted cardioverter defibrillators demonstrating an association between PM_2.5_ elevation and increased incidence of ventricular arrhythmias and rapid atrial fibrillation suggest autonomic dysregulation as a putative pathway to these clinical end points (Dockery et al. 2005; Peters et al. 2000; Rich et al. 2006). The role of PM_2.5_-mediated autonomic dysregulation has been explored using heart rate variability (HRV) as a surrogate for autonomic tone (Task Force of the European Society of Cardiology 1996) in epidemiologic studies (Creason et al. 2001; Gold et al. 2000; Liao et al. 1999; Magari et al. 2001), animal studies (Godleski et al. 2000), and human experimental studies (Devlin et al. 2003; Gong et al. 2003). Yet there is still a scarcity of knowledge about health effects of potential synergy between PM_2.5_ and ambient gaseous copollutants (National Research Council 2004). Of these copollutants, O_3_ interaction is of particular interest given its possible role in local oxidation, creation of free radicals, and potentiation of pollutant deposition onto surfaces (Finlayson-Pitts and Pitts 1997; Meng et al. 1997).

To assess whether the association between particulate exposure and autonomic function is altered by concomitant O_3_, we conducted a randomized, single-blind, crossover study, with subjects undergoing exposure to concentrated ambient particles (CAPs), O_3_, combined CAPs and O_3_, and filtered air. Secondary aims of the study included assessing for a dose–response relationship for CAPs exposure both with and without O_3_, assessing blood pressure (BP) response to exposures, and assessing whether a subgroup of subjects with asthma was more susceptible to autonomic dysregulation after exposures.

## Materials and Methods

### Study population

The study was approved by the Human Subjects Review Committee of the University of Toronto and was performed at the Gage Occupational and Environmental Health Unit in Toronto, Ontario, Canada. Informed consent was obtained from 50 male and female nonsmoking subjects, 19–48 years of age, without any history of cardiovascular disease or risk factors. Subjects were pooled from two separate studies, study A and study B. One subject participated in both studies.

From July 1999 through February 2003, study A conducted exposures on subjects with and without asthma (*n* = 21). Asthmatic status was determined by physician diagnosis and confirmed by methacholine challenge. Asthmatic subjects had been off of any short-acting bronchodilators from midnight onward and off of corticosteroid treatment for at least 1 month before all visits. Study B was conducted from January 2004 through October 2007, and included only subjects without asthma (*n* = 30). BP responses to exposure in study B have been reported previously (Urch et al. 2004).

### Exposure protocol

All subjects were intended to receive four 2-hr exposures in random order as follows: *a*) sham filtered air (FA) with particles removed by a high-efficiency particulate arrester (HEPA) filter and no added O_3_; *b*) CAPs with target PM_2.5_ mass concentration of 150 μg/m^3^; *c*) O_3_ with target concentration of 120 ppb; and *d*) CAPs + O_3_. In addition, a subset of participants in study A underwent additional exposures with a target CAPs mass concentration of 60 μg/m^3^. Exposures within the same subject were separated by at least 2 weeks, and all exposures were conducted at the same time of day to avoid confounding by circadian variations (Brook et al. 2002; Task Force of the European Society of Cardiology 1996). In addition, subjects were seated at rest in the exposure chamber 15 min before receiving the intended exposure to allow for acclimation to sitting in the chamber.

Details of the human exposure facility have been described previously (Brook et al. 2002; Petrovic et al. 2000; Urch et al. 2005). Briefly, ambient particles with aerodynamic diameter < 2.5 μm were drawn through an inlet outside of the laboratory, concentrated using a Harvard virtual impactor (Sioutas et al. 1995), diluted to the target concentration, and delivered via facemask to subjects seated at rest inside an enclosure (Petrovic et al. 2000). The achieved CAPs mass concentration was measured gravimetrically from samples obtained via a port proximal to the facemask (Petrovic et al. 2000). The concentrator was run during the non-CAPs exposures, but particles were removed with a HEPA filter proximal to the facemask (Petrovic et al. 2000; Urch et al. 2004). O_3_ was produced with an arc generator using medical-grade oxygen and delivered upstream of the particle concentrator. O_3_ concentration was maintained at approximately 120 ppb measured just upstream of the facemask by an O_3_ analyzer (model 1008-RS; Dasibi Environmental, Glendale, CA, USA). Ambient temperature, relative humidity, and ambient gaseous pollutant concentrations including carbon monoxide, carbon dioxide, sulfur dioxide, nitrogen oxide, and nitrogen dioxide were monitored during the exposure as previously described (Petrovic et al. 2000; Urch et al. 2004). In addition, CAPs mass concentration was continuously monitored using a tapered element oscillating microbalance monitor (TEOM) (model 1400a; Rupprecht & Patashnick Co. Inc., Albany, NY, USA).

### Outcome measurement

**Figure 1:**
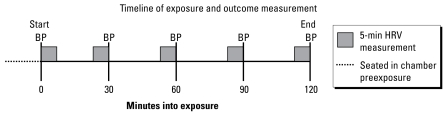
Timeline of BP and HRV measures.

The primary outcome measure was change in several HRV indices between the start and end of each 2-hr exposure (Figure 1). Electrocardiograms were continuously recorded using digital Holter monitors (Marquette Medical Systems, Milwaukee, WI, USA) and subsequently stored and analyzed on a Marquette Medical Systems (MARS) Unity workstation. Beat annotations were automatically assigned by the software and reviewed by an investigator blinded to the exposure status.

Calculations for time domain [standard deviation of NN intervals (SDNN); square root of the mean squared differences of successive NN intervals (rMSSD); proportion of successive NN intervals with differences > 50 msec (pNN50)] and frequency domain [low-frequency (LF) power (0.04–0.15 Hz), high-frequency (HF) power (0.04–0.15 Hz), and their ratio (LF/HF)] HRV parameters were evaluated on 5-min intervals of ECG data using standard techniques (Task Force of the European Society of Cardiology 1996). Only normal sinus beats were used. These measures were calculated at 0, 30, 60, 90, and 120 min into the exposure (Figure 1). For intervals with < 90% valid quality recording, the previous 5-min window was used instead.

In addition, systolic blood pressure (SBP) and diastolic blood pressure (DBP) were measured every 30 min throughout the 2-hr exposure (Figure 1) using a calibrated and automated sphygmomanometer (Oscar-1 or Oscar-2; SunTech Medical Instruments, Inc., Raleigh, NC, USA). Where the data were available, the analysis used a mean of three successive BP measurements at each time point. We also included mean arterial pressure (MAP) in our measurements, defined as the weighted average (2:1) of DBP and SBP. Furthermore, minute ventilation and respiratory frequency were recorded every 30 min during the exposure using a turbine-type flow transducer (VMM401; Interface Associates, Aliso Viejo, CA, USA).

### Statistical analysis

We used linear mixed-effects models to evaluate the effects of exposure while accounting for the correlation between multiple measurements within each individual. In a first analysis, models included fixed effects for exposure (in four categories: FA, CAPs, O_3_, and CAPs + O_3_), age (linear continuous), sex, and asthmatic status (yes/no), and a random-subject intercept, modeling the primary outcome of change in HRV or BP between start and end of exposure as linear continuous variables. In a second analysis, we entered average measured CAPs mass concentration into the model as a linear continuous-exposure variable and stratified the CAPs dose–response analysis by presence or absence of O_3_.

We conducted several sensitivity analyses to evaluate the robustness of our main results. First, to assess whether additional time points of HRV and BP data would improve the resolution of the models, we modeled the mean change in outcome across five measurements taken every 30 min during the 2-hr exposure. Second, we evaluated whether the responses to CAPs and O_3_ differed in asthmatics versus non-asthmatics by adding exposure-by-asthmatic status interactions to the main model. Third, we controlled for potential confounding by minute ventilation and respiratory frequency, which can especially affect HF HRV (Hayano et al. 1991; Task Force of the European Society of Cardiology 1996). Fourth, we added average heart rate from the prior 5-min interval as a fixed effect.

All hypothesis tests were two-sided, and *p* < 0.05 was considered statistically significant. We used the SAS statistical analysis package (version 9.1; SAS Institute, Cary, NC, USA) for all analyses. Regression diagnostics were performed to verify model assumptions.

## Results

### Subject characteristics and exposures

**Figure 2:**
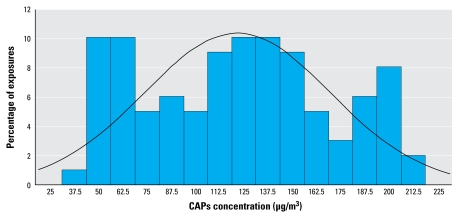
Percentage distribution of CAPs exposure concentrations. The line indicates the fitted normal density curve.

A total of 50 subjects with complete Holter data were included from both studies in the analyses (Table 1). The main distinction between study A and study B was the inclusion of 10 mildly asthmatic subjects in study A. The number of exposures with complete Holter data in each exposure category was as follows: 53 exposures to CAPs; 53 exposures to CAPs + O_3_; 40 exposures to O_3_; and 42 exposures to FA (sham). By design, a subset of participants in study A underwent additional exposures to CAPs at a lower target concentration, resulting in relatively fewer FA- and O_3_-only exposures. The mean CAPs mass concentration was 121.6 ± 48.0 μg/m^3^ (Figure 2). The mean O_3_ concentration was 113.9 ± 6.6 ppb. During FA exposure, the mean CAPs mass and O_3_ concentrations were 2.3 ± 5.7 μg/m^3^ and 10.0 ± 7.4 ppb, respectively.

### Outcomes

#### Heart rate variability

**Figure 3:**
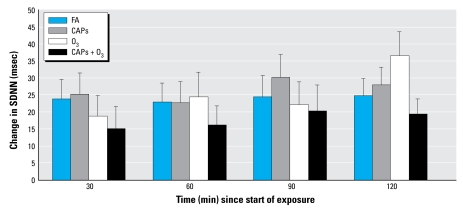
Change in SDNN by exposure from baseline over 2 hr. Error bars indicate SE.

As illustrated in Figure 3, for all of the exposures, subjects had a net increase in SDNN as the 2-hr exposure progressed. The trend with time was similar for the other indices of HRV. The primary analysis, change in HRV indices between the start and end of the 2-hr exposure period, yielded no consistent differences between the exposure categories (Table 2). HF HRV showed a statistically significant increase for the CAPs-only exposure (*p* = 0.046) and a similar trend for O_3_ exposure (*p* = 0.051) when compared with filtered air. Sensitivity analyses separately incorporating minute ventilation, respiratory frequency, average heart rate, or the asthma interaction term into the main model abolished this difference and did not materially alter findings for the other HRV outcomes. Analyses using the mean of all five HRV measurements during the 2-hr exposure or assessing change in HRV by minutes into exposure did not materially alter our conclusions. We did not find evidence of heterogeneity by asthmatic status.

We also analyzed the dose–response relationship between CAPs mass concentration and change in the HRV indices, stratified by presence or absence of O_3_. For those exposures without O_3_ (i.e., CAPs alone and FA), there was no significant dose–response relationship between gravimetric CAPs mass concentration and any measure of HRV. However, when analyzing the CAPs mass concentration relationship for exposures with O_3_ (i.e., CAPs + O_3_ and O_3_ alone), there was a suggestion of negative dose–response slopes (Table 3) between CAPs mass concentration and several HRV indices. This trend was seen in SDNN, rMSSD, and HF and was statistically significant for LF (*p* = 0.02).

#### Blood pressure

A statistically significant increase in DBP for CAPs + O_3_ relative to FA was previously reported for the subjects in study B (Urch et al. 2005). Therefore, in the current analysis, we focused only on the BP data from study A.

**Figure 4:**
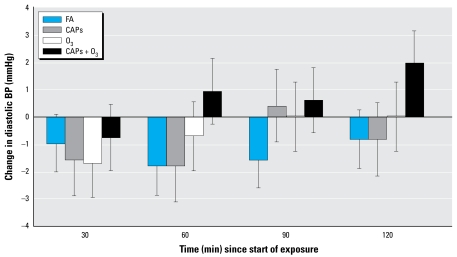
Change in DBP over 2 hr by exposure. Error bars indicate SE.

We assessed the change in BP between the start and end of the the 2-hr exposure (Table 4) as well as the change in BP at each 30-min interval. Similar to data from study B, subjects in study A demonstrated a statistically significant increase in DBP (1.97 mmHg, *p* = 0.02) in response to CAPs + O_3_. Figure 4 shows DBP at 30-min intervals throughout the exposure period. All the subjects experienced an equilibration period after entering the chamber when their DBP decreased initially with time. After this equilibration, the CAPs + O_3_ exposure began to diverge from the other exposures, demonstrating a statistically significant increase relative to filtered air at 60 min (*p* < 0.03). No clear pattern emerged among the exposures for SBP and MAP, although SBP tended to decrease in the O_3_-only exposure (Table 4).

Sensitivity analyses additionally controlling for minute ventilation, respiratory frequency, or heart rate did not materially alter the results. We did not find evidence of heterogeneity of effect by asthmatic status.

## Discussion

To our knowledge, this is the largest study assessing the acute impact of CAPs exposure on HRV in young healthy adults. In addition, with recent evidence in animals showing effects of combined O_3_ and particulate exposure on HRV (Hamade et al. 2008), this is also the largest human controlled-exposure study looking at the impact of combined exposure to CAPs and O_3_ on HRV.

In this study, HRV indices were used as surrogate markers of autonomic tone (Task Force of the European Society of Cardiology 1996). The time-domain measures rMSSD and pNN50 are thought to reflect parasympathetic influences, whereas SDNN may also have sympathetic influence (Dekker et al. 2000; Hayano et al. 1991; Task Force of the European Society of Cardiology 1996). Among the frequency-domain parameters, HF almost exclusively reflects parasympathetic influence and respiratory variation (Hayano et al. 1991; Task Force of the European Society of Cardiology 1996). Interpreting changes in LF and LF/HF is controversial; LF may to some extent reflect sympathetic influences, whereas LF/HF possibly reflects sympathovagal balance (Task Force of the European Society of Cardiology 1996).

There is a large body of evidence suggesting that cardiovascular response to pollutants varies with age and comorbidities (Brook et al. 2004; Dockery et al. 1993; Goldberg et al. 2001; Katsouyanni et al. 2001). In this group of young healthy adult subjects, we did not detect a consistent pattern for changes in HRV indices among the exposure categories. HF had a statistically significant difference in some of the exposures relative to filtered air. However, given that HF is particularly sensitive to respiratory variation (Task Force of the European Society of Cardiology 1996), adjusting for respiratory parameters abolished this difference. Despite the absence of a clear pattern of difference in the categorical exposure models, the dose–response analysis in the setting of concomitant O_3_ demonstrated a trend toward negative linear association between CAPs mass concentration and change in several HRV indices, with a statistically significant relationship for LF HRV. No such relationship existed without O_3_ present. Our findings for CAPs alone agree with findings in other studies reporting the lack of significant effect of CAPs alone on HRV in young healthy subjects, despite significant effects noted in elderly subjects (Devlin et al. 2003; Gong et al. 2003). The suggestion of dose–response CAPs effects in the presence of O_3_ is of import, given emerging research priorities emphasizing studies of copollutants and dose–response relationships (National Research Council 2004).

Our results corroborate previous findings of increase in DBP with CAPs + O_3_ (Urch et al. 2005). The effect became statistically significant at 60 min and became more pronounced toward the end of the exposure. With regard to SBP, only one human controlled-exposure study (Gong et al. 2003) has demonstrated modest changes with CAPs exposure, whereas other studies have not shown effects (Devlin et al. 2003; Urch et al. 2005). In one exposure study conducted in canines exposed to CAPs, the increase in DBP was of greater magnitude than the increase in SBP (Bartoli et al. 2009). One possible explanation for the apparent disparity between SBP and DBP change in our study is the attenuation of peripheral SBP amplification in young adults (Franklin et al. 2005). It has been shown in adults < 50 years of age that for a given increase in central and peripheral DBP, peripherally measured SBP does not increase in parallel as it does for older adults (Wilkinson et al. 2001). Alternatively, the baseline increase in SBP in our filtered-air control group and the greater variation and lability of SBP measurements relative to DBP in our study may have made it more difficult to appreciate a meaningfully different change in SBP.

The effects of CAPs + O_3_ on BP, and possibly in a dose–response fashion on HRV, raise important questions about the interaction and possible synergy between the two components in their impact on cardiovascular physiology. With regard to possible mechanisms for pollutant-mediated changes in systemic physiology, it has been demonstrated in animal models that O_3_ exposure leads to lung hyperpermeability (Kleeberger et al. 2000), possibly increasing systemic toxin absorption or increasing local inflammation. Other possibilities include O_3_-mediated free radical production or conversion of particles to compounds with greater tendency to deposit on surfaces, the latter having been demonstrated with ammonium nitrate particles (Meng et al. 1997). In humans, generated ammonium nitrate particle with O_3_ combination exposure has recently been shown to suppress HRV (Power et al. 2008). The changes in DBP may be related to changes in autonomic tone, as shown in cohort studies demonstrating correlation among HRV measures and incident hypertension (Singh et al. 1998), but the data in our study do not consistently validate this hypothesis. Alternatively, the DBP response may be related to direct vasoconstriction (Brook et al. 2002; Urch et al. 2004, 2005) or increased peripheral vascular resistance independent of autonomic changes, but we did not directly test this hypothesis in this study.

Our study has several potential limitations that need to be considered. First, we could not control for ambient pollutant exposure prior to each visit. Future studies may address this limitation with personal monitoring, detailed time–activity diaries, or optimally, having study participants sleep onsite the night preceding any experiments. Second, because the study was designed to examine acute effects, we could not assess more prolonged time-dependent effects of exposures (McDonnell et al. 2007) or effects resulting from sequential rather than concomitant exposures. Future studies may address this limitation using staggered and/or prolonged exposures. Third, because our particle concentrator does not concentrate particles < 0.1 μm, limiting particle size to predominantly accumulation mode, we could not assess the role of ultrafine particles. Controlled-exposure studies comparing the toxicity of ultrafine, fine, and coarse particles in humans are clearly needed. Fourth, we did not measure or remove volatile organic compounds, which likely have independent effects (Bhatnagar 2006). However, the crossover design of this study reduced potential confounding either by exposure variation or by other unmeasured confounders.

An important strength of this study is that CAPs exposures were derived from real-world ambient particles from an urban environment. According to one sampling campaign in 1997 through 1999, PM_2.5_ in Toronto typically includes approximately one-quarter organic carbon, similar portions sulfates, nitrates, ammonium, and black carbon, and small portions of soil or other constituents (Bhatnagar 2006). The large sample size of our study and the fact that composition of CAPs changes daily in these experiments can be exploited in future analyses to identify components or constituents of CAPs that may be associated with greater effects.

## Conclusion

In summary, ambient PM_2.5_ has been shown to impact HRV in epidemiologic studies of populations considered vulnerable on the basis of age or chronic diseases (Creason et al. 2001; Gold et al. 2000; Liao et al. 1999; Magari et al. 2001; Pope et al. 1999). Whereas most epidemiologic studies focus on effects ranging from several hours to days (Creason et al. 2001; Liao et al. 1999; Magari et al. 2001; Pope et al. 1999), we attempted to study acute effects within the span of 2 hr. Our data suggest that in younger subjects, CAPs alone does not produce significant effects, but when combined with O_3_, there is an increase in DBP that may be mediated through autonomic changes. The possibility of an autonomic effect is suggested in the dose–response relationship of HRV to CAPs mass concentration, although this is of small magnitude and borderline statistical significance in this group of young healthy adult subjects. Given that CAPs alone produce HRV changes in more vulnerable populations (Creason et al. 2001; Devlin et al. 2003; Gold et al. 2000; Liao et al. 1999; Magari et al. 2001; Pope et al. 1999), elderly subjects or subjects with comorbidities may experience even stronger synergistic effects with simultaneous controlled exposure to CAPs and O_3_.

## Figures and Tables

**Table 1:** Baseline subject characteristics and exposure data.

Characteristic	Overall	Study A	Study B
No. of subjects	50^a^	21	30
Age [years, mean ± SD (range)]	27.08 ± 7.13 (19–48)	27.29 ± 5.60	26.93 ± 8.13
Male [no. (%)]	24 (47)	9 (43)	15 (50)
Ethnicity [no. (%)]			
White	24 (49)	10 (48)	14 (50)
Black	7 (7)	4 (19)	3 (11)
Asian	16 (33)	6 (29)	10 (36)
Other	2 (4)	1 (5)	1 (4)
Body mass index [kg/m^2^ (mean ± SD)]	22.72 ± 3.24	22.35 ± 2.47	23.22 ± 3.54
Medical history		10 Asthmatics	None
CAPs [μg/m^3^ (mean ± SD)]	127.03 ± 62.13	101.16 ± 42.43	146.20 ± 67.58
O_3_ [ppb (mean ± SD)]	113.86 ± 6.62	118.69 ± 3.31	110.30 ± 6.19

a One subject was included in both studies.

**Table 2:** Change in HRV (± SE) between start and end of 2-hr exposure period.

HRV variable/Exposure	ΔPre- and postexposure	*p*-Value^a^
Δ5-Min average heart rate (bpm)		
FA	2.57 ± 0.91	
CAPs	2.09 ± 0.93	0.61
O_3_	1.55 ± 0.95	0.28
CAPs + O_3_	2.04 ± 1.09	0.62
ΔSDNN (msec)		
FA	22.14 ± 4.87	
CAPs	26.31 ± 5.10	0.41
O_3_	34.29 ± 7.30	0.098
CAPs + O_3_	17.74 ± 4.29	0.31
ΔrMSSD (msec)		
FA	−4.70 ± 6.28	
CAPs	5.51 ± 7.74	0.19
O_3_	16.77 ± 11.92	0.07
CAPs + O_3_	−0.12 ± 6.79	0.50
ΔpNN50 (%)		
FA	−1.48 ± 2.01	
CAPs	0.25 ± 1.74	0.32
O_3_	1.28 ± 2.32	0.24
CAPs + O_3_	−1.18 ± 2.33	0.90
ΔLF (msec^2^)		
FA	456 ± 449	
CAPs	1381 ± 671	0.17
O_3_	1915 ± 1167	0.21
CAPs + O_3_	482 ± 474	0.96
ΔHF (msec^2^)		
FA	−149 ± 198	
CAPs	348 ± 247	0.046
O_3_	467 ± 312	0.051
CAPs + O_3_	21 ± 239	0.48
ΔLF/HF ratio		
FA	0.73 ± 0.45	
CAPs	0.62 ± 0.55	0.84
O_3_	0.52 ± 0.56	0.70
CAPs + O_3_	0.12 ± 0.62	0.33

a Compared with exposure to filtered air.

**Table 3:** Dose–response slope for CAPs mass concentration versus 2-hr change in HRV.

HRV measure	Exposure	Slope^a^	SE	*p*-Value^b^	*p*-Contrast^c^
Δ5-min average heart rate (bpm)	No O_3_	−0.033	0.060	0.58	
	With O_3_	0.014	0.057	0.80	0.56

ΔSDNN (msec)	No O_3_	−0.0033	0.39	0.99	
	With O_3_	−0.84	0.37	0.03	0.12

ΔrMSSD (msec)	No O_3_	0.38	0.59	0.52	
	With O_3_	−0.96	0.56	0.09	0.10

ΔpNN50 (%)	No O_3_	0.08	0.13	0.53	
	With O_3_	−0.13	0.12	0.28	0.23

ΔLF (msec^2^)	No O_3_	80.38	55.71	0.15	
	With O_3_	−94.51	52.53	0.074	0.02

ΔHF (msec^2^)	No O_3_	18.97	17.57	0.28	
	With O_3_	−22.19	16.63	0.18	0.09

ΔLF/HF ratio	No O_3_	0.0048	0.034	0.89	
	With O_3_	−0.034	0.032	0.29	0.41

a Slopes reported for a 10-μg/m^3^ change in CAPs mass concentration.

b *p*-Homogeneity.

c *p*-Contrast between slope with O_3_ and slope without O_3_.

**Table 4:** Change in BP between exposure start and exposure end.

BP measure/exposure	ΔBP ± SE	*p-*Value^a^
ΔSBP (mmHg)		
FA	2.32 ± 1.79	
CAPs	0.04 ± 2.51	0.37
O_3_	−1.18 ± 1.96	0.07
CAPs + O_3_	0.90 ± 2.00	0.48
ΔDBP (mmHg)		
FA	−0.84 ± 1.20	
CAPs	−0.87 ± 1.43	0.98
O_3_	−0.23 ± 1.57	0.70
CAPs + O_3_	1.97 ± 1.21	0.02
ΔMAP		
FA	0.17 ± 1.21	
CAPs	−0.62 ± 1.65	0.63
O_3_	−0.56 ± 1.48	0.62
CAPs + O_3_	1.64 ± 1.15	0.20

a Compared with exposure to filtered air.
